# On scientific lectures with evaluation committee blessings

**DOI:** 10.1016/S1808-8694(15)31255-6

**Published:** 2015-10-20

**Authors:** Henrique Olival Costa

For a long time, the scientific community has demonstrated uneasiness with what is said and written in the academic community. It seems that a skeptical wave emerges from the democratization and consequent demystification of the media, among them, scientific journals.

Currently, the possibility of bias is common, as is lack of investigative strictness and even lack of veracity in many worldwide magazines. The conflict of interest, when peers participate in projects whose results, positive or not, favor a company where the researcher is employed or has any liaison or benefit, and the need of scientific production generated by the academic system, in which those who publish the most earns more, lead to uncountable clone works with solely one research as main source. Moreover, the rising of apocryphal “spokesman” media of commercial companies, that publish articles with a content slightly beyond scientific interest, have brought about a weakness in the reliability of reading material. In a certain way, this kind of incredulity may be extremely healthy as it extracts the dogmatism present in the written word and that old feeling of what is in writing has to be true, especially when endorsed by an international publication database, beginning to experience significant changes. This exceptional change condition reveals that, as everything in life, both sides should be considered: first is favoring and widening the actions of the scientific-literary review, as the possibility of any professional to read, absorb, criticize and reflect over each of the 8,000 or more medical publications published each year would be unthinkable.

The rising of professionals with scientific commonsense, research knowledge and the analytical skills to analyze and convey opinion on the main media and its publications begins to shape up. In the same way, the valorization of a journal’s acceptance index, by impact and quote factors, shows “who is who” in the ruthless competition for a place in the sun in the medical scientific publishing universe.

It is obvious that, despite the improvements, incoherence occurs in such cases as with inappropriate sampling of the main indexes used, such as the ISI, given that very few publications are indexed on JCR, from where factor source is obtained, and therefore, present a sampling bias. Anyway, the bright side of the skeptical pessimism would be, in the med-term, the appreciation of quality instead of quantity. The dark side of the situation would be the progressive denial of the media with the reduction of its reliability. Probably both sides will tend to come closer, what has been actually occurring, in a certain way, with the natural selection of magazines, being the survivors those with the highest demand, probably because they present more scientific value (at least from the reader’s point of view) and the rising of journals specialized in searching for impacting articles in several areas of knowledge. Adding this to the several institutes specialized in the production of systematic review of the literature brings us through which we could renew the credibility of scientific journals, while at the same time favoring sound and rigorous research.

But it is important to remember that not all professionals have the time or disposition, or even the skills, to collect useful information from scientific articles. We know that much of this information is hidden by methods and statistical tricks, which are not always at reach, or maybe are not noticed during distracted reading. For this group of “excluded souls”, due to lack of time, lack of concentration or education, a safe substitute must be conceived.

Nowadays we consider this to be the role of medical school professors and specialized congress lecturers. We have observed that we barely take classes where the lecturer does not present any bibliographical reference when controversial concepts or novelties are mentioned. This is a healthy measure and fosters knowledge dissemination in a more critical manner. However, the use of references is expected to be made with criteria and limits, and besides, that everyone does it, standardizing lectures and changing our culture of informal knowledge, where concepts are expressed and propagated, without mentioning their source, creation process or validation. This is where Congresso Triologico de Otorrinolaringologia could set a trend.

The change of lecturing invitations into a kind of free-paper evaluation could help build this eminently scientific and rigorous tendency to presentations. A system with this characteristic, besides guaranteeing deeper care in the preparation of presentations, could help standardize their format and minimum requirements, besides being the possible way in to the academic life for many promising young people who sometimes have their introduction delayed because of their distance to congress decision circles, as they are neither in large schools nor in build-up education centers.

During the last three Brazilian and Triologico Congresses we have been progressively improving the format of free communication analysis, in order to standardize it and adequate it to a scientific accreditation policy.

Today an evaluation system based on the platform of the Brazilian Journal of Otorhinolaryngology is in use, using the online system and the reviewers of our journal. We also foster the basic scientific assumptions for the investigation process chosen by our Magazine. In a certain way, this has professionalized the inner workings of the projects and made feasible the evaluation to be executed by three judges, in a double-blind format, for each topic sent.

The strictness level is yet not the same as in the journal, as goals are different, but it is aiming to that direction, at least in what concerns oral presentations that, in the Triologico Congress, tend to occupy even further the prime time of the events. Currently the evaluation process is divided into four steps: in the first one, the formats of projects are evaluated so that they fulfill all RBORL submission requirements. Projects must be complete, with results, discussion and conclusions duly clear and a structured abstract must be presented. Once the requirements are fulfilled, the work’s content is evaluated and if there is a scientific question, it will be judged for oral presentation and will be eligible to an award. If it is a project of investigation of series of cases or a rare case description, it will be forwarded to be presented as poster. After the presentation format is defined, if approved, the work is evaluated in regards to the theme area it is included, so that the judges qualified in that area are allocated.

At first, every work receives three opinions, double-blind, where the topic must be evaluated regarding the impact on the specialty, methodological appropriation, results’ transparency, discussion properness and conclusion consistency. In addition to the grade received, all projects must be approved or not for Congress presentation. The grade average will define if the project is within the top 10 best of the specialty area and if it will run for the award of “Best Scientific Work – BSW”, if the work will be presented orally at the Congress, if it will be presented as a poster or if it will be refused. Finally, during Congress, all works presented, in any format, will be judged by an in-house committee, and the BTW awards, best young researcher project and best poster will be announced.

For this task to be completed, an army of skilled reviewers experienced in scientific review is necessary, recruited from the publishing team of the Brazilian Journal of Otorhinolaryngology and along with the co-workers that have already gone through this process of scientific approval during post-graduation, having, at least, a master degree or their PhDs. Thus, we consider we reached an excellent judgment standard that is perfected during the process, given that there are preparatory meetings to determine the level of requirements and the operational procedures of the evaluation process at the event’s site.

In the current process, 100 judges were recruited for the first two steps and another 100 will be called for the Congress judgment.

Four hundred papers were submitted and approved, several with very high scientific standards, contributing to our event’s quality. The winner is the specialty, the winner is the specialist.

Having said that, I’d like to thank all judges, reviewers and committee members who participated in the previous selection process, in-house evaluation and free-paper discussion of oral presentation or posters of our Triologico Congress, saying that they already are and will always be fully responsible for the seriousness and quality of our scientific events.

Regards,

